# Epidemiological Characteristics and Influencing Factors of Myopia Among Primary School Students in Southern China: A Longitudinal Study

**DOI:** 10.3389/ijph.2023.1605424

**Published:** 2023-02-14

**Authors:** Jingfeng Mu, Dan Zeng, Jingjie Fan, Meizhou Liu, Mingjie Jiang, Xinyi Shuai, Jiantao Wang, Shaochong Zhang

**Affiliations:** ^1^ Shenzhen Eye Hospital, Jinan University, Shenzhen Eye Institute, Shenzhen, China; ^2^ Shenzhen Maternity and Child Healthcare Hospital, Southern Medical University, Shenzhen, China

**Keywords:** prevalence, longitudinal study, myopia, schoolchildren, influencing factor

## Abstract

**Objectives:** To study the epidemiological characteristics and influencing factors of myopia to provide a scientific basis for the prevention and control of myopia.

**Methods:** 7,597 students studying in grades 1–3 were followed up. Eye examinations and questionnaire surveys were conducted annually from 2019 to 2021. The influencing factors of myopia were analyzed by logistic regression model.

**Results:** The prevalence of myopia among students in grades 1–3 in 2019 was 23.4%, which increased to 41.9% and 51.9% after the 1-and 2-year follow-up, respectively. The incidence of myopia and change in the spherical equivalent refraction (SER) were higher in 2020 than in 2021. The 2-year cumulative incidences of myopia were 2.5%, 10.1%, 15.5%, 36.3%, and 54.1% in students with a baseline SER >+1.50D, +1.00D to +1.50D, +0.50D to +1.00D, 0.00D to +0.50D, and -0.50D to 0.00D, respectively. Outdoor activities, sex, age, baseline SER, parental myopia, sleep time, and digital device exposure were associated with myopia.

**Conclusion:** The prevalence of myopia demonstrated a rapid increase; thus, healthy habits and outdoor activities should be promoted for the prevention and control of myopia.

## Introduction

Myopia is a global public health problem (1). The prevalence of myopia among school-age children is increasing, especially in East and Southeast Asia (2). Holden (3) predicted 4.758 billion individuals to have myopia in the world by 2050, accounting for approximately 49.8% of the total population. Studies reported the prevalence of myopia among students in grade 1 in primary and junior middle schools in China to be 3.9% and 67.3%, respectively (4). With the increasing prevalence of myopia, uncorrected refractive error has become an important cause of visual impairment (5). By 2050, approximately 100 million Chinese people may suffer irreversible vision loss and become blind owing to the high prevalence of myopia in China (2). Myopia, especially high myopia, can cause many complications that harm visual health and even result in blindness (6). High myopia-related retinopathy is the leading cause of irreversible blindness in adults in some areas of China (7). Moreover, vision loss is a huge economic burden for individuals worldwide (8), and the global economic cost of visual impairment attributed to uncorrected myopia is $244 billion annually (9).

The causes of myopia are complex. A twin study demonstrated myopia to be highly heritable**,** thereby suggesting a genetic predisposition (10). Compared to children whose parents have normal vision, children whose parents are myopic possess a significantly increased risk of myopia (11). A major change has not been observed in Genetics and patterns of inheritance in recent years. However, the prevalence of myopia worldwide is still increasing significantly, indicating the role of environmental factors in the development of myopia (12). Environmental factors such as outdoor activities (13), educational level (14), electronic devices (15), sleep time (16), and reading and writing posture (17) are associated with myopia.

Myopia, which is the most common visual impairment among children and adolescents (18), bears serious health risks and financial burden. Owing to the lack of effective treatment methods, understanding the epidemic characteristics, occurrence, development, and influencing factors of myopia is critical to explore and implement effective preventive and control measures. Currently, many cross-sectional studies exist on the prevalence of myopia among school students (19–21), however, few longitudinal studies have examined myopia progression among school students in China. Longitudinal studies on the progression of myopia were carried out in Chongqing, Southwest China, in 2006 (22); Guangzhou, Southern China, in 2010 (23); and Anyang, Central China in 2012 (24). These studies explored the principle of changes in the students’ refraction status but did not investigate and analyze the influencing factors. In addition, these studies were conducted 10 years ago; hence, and it is possible that the principles of myopia progression in the students may have changed. Cohort studies on the progression of myopia have also been conducted in other countries, such as Australia (25) and Northern Ireland (26). However, these studies lacked annual follow-up monitoring data, and the follow-up samples were small.

Currently, there is a lack of recent large-scale longitudinal studies among school students in South China. A longitudinal study can reveal the principles of disease occurrence and development and explore the cause of the disease. In 2019, we conducted the Shenzhen Child and Adolescent Eye Study (21). Based on that study, we conducted a longitudinal study to explore the epidemiological characteristics of myopia among school-aged children and explore the factors influencing myopia. We aimed to provide a theoretical basis for the prevention and control of myopia in this study.

## Methods

### Ethics Statements

This study was approved by the Ethics Committee of Shenzhen Eye Hospital. The students and their parents or guardians were informed of the objectives and examination procedures of this study, and written informed consent was obtained from the parents or guardians.

### Study Design and Population

Shenzhen is located in Southern China and consists of 10 administrative districts. This study was conducted using cluster sampling. One primary school was randomly selected from each administrative district in Shenzhen by a random number, and all the students in grades 1–3 of the selected schools participated in this study. The sample size was calculated using the G*Power software (version 3.1.9.7), which can compute effect sizes (27). According to the G*Power software, the sample size of the students should be 7,220. Considering the possibility of loss to follow-up in the follow-up study, 9,153 students were included in this study for follow-up.

The inclusion criteria were as follows: 1) Students whose parents or guardians agreed to participate in this study, 2) Students who were in grades 1–3 in 2019, and 3) Students who completed all the items in the three inspections from 2019 to 2021. The exclusion criteria were as follows: 1) Students who were unable to undergo the visual acuity test, refraction test, and questionnaire survey, 2) Students with eye disease, and 3) Students who wore orthokeratology lenses. In total, 9,153 students in Shenzhen were enrolled in the study. 8,241 students completed the 1-year follow-up, and 7,597 students completed the 2-year follow-up. The reasons for loss to follow-up were transfer to another school, requesting leave and opting not to undergo the tests. After the 2-year follow-up, 591, 92, and 873 students in grades 1, 2, and 3 were lost to follow-up, respectively. Further analysis revealed that there was no difference in the sex and baseline spherical equivalent refraction (SER) between the lost to follow-up population and the follow-up population. A total of 2,409 students from grade 1 (1,299 boys and 1,110 girls), 2,713 students from grade 2 (1,464 boys and 1,249 girls), and 2,475 students from grade 3 (1,360 boys and 1,115 girls) were followed up from September 2019 to September 2021. Visual acuity and refraction tests were conducted by trained doctors and nurses in September 2019, September 2020, and September 2021 in this study. The questionnaire survey was conducted by trained doctors in September 2021 in this study.

### Visual Acuity Test

The visual acuity test was performed in accordance with the specifications for screening for refractive error among school students (28). Visual acuity tests were performed by optometrists using an electronic visual acuity chart (Eye Vision 1603-01) in a bright classroom. Eye mask plates were used to cover the contralateral eye during the examination, and the right eye was examined first before the left eye. In order to ensure the accuracy of the examination, the students were required to sit in their seats and look straight ahead to correct their behavior of leaning forward and squinting at any time. Visual acuity lower than 5.0 in either eye was considered abnormal visual acuity, and visual acuity ≥5.0 in both eyes was considered normal visual acuity.

### Refraction Test

The refraction test was conducted in accordance with the specifications for the screening for refractive error among school students (28). Non-cycloplegic refraction tests were performed by optometrists using an autorefractor (NIDEK AR-1). The refractive test was automatically repeated three times for every student, and the average values of SER of the participants were obtained.

### Questionnaire Survey

The questionnaire was compiled by numerous experts according to domestic and foreign questionnaires concerning myopia. The questionnaire was completed by the students’ parents or guardians. The questionnaire collected the students’ general information and habits associated with eye health, including but not limited to age, sex, time of birth, parental myopia, education, time spent performing outdoor activities, digital device exposure, sleep time, family average monthly income, and living space.

### Definition of Myopia

Myopia was defined as SER <−0.50 diopter (D) tested by non-cycloplegic refraction and uncorrected visual acuity <5.0 (28). According to SER, subjects with myopia were divided into three groups in this study: mild myopia (−0.50 D ∼ −3.00 D), moderate myopia (−3.00 D ∼ −6.00D) and high myopia (≤−6.00D).

### Statistical Analyses

SER follows a normal distribution according to the Kolmogorov–Smirnov test; SER is expressed as the mean and standard deviation. Independent samples t-tests were performed to compare the means between groups, and repeated measures analysis of variance was used to estimate the effect size. Qualitative data are presented as frequency and percentage, and Chi-square tests were performed to compare the percentages between the groups. Pearson’s correlation analysis revealed that the SERs of the left and right eyes were correlated (*r* = 0.80, *p* < 0.001), and the SERs of the right eyes were used to evaluate the epidemiological characteristics of myopia in this study. The incidence of myopia in this study was calculated as the number of new myopia cases during the follow-up period divided by the number of students who were not myopic at the beginning of the follow-up, and the prevalence of myopia was defined as the number of students with myopia divided by the number of students during the observation period. Logistic regression was conducted to analyze the factors of the incidence of myopia, and odds ratios (ORs) and 95% confidence intervals (95% CIs) were provided*.* Logistic regression models were used to analyze the influencing factors of myopia. We first conducted a simple model analysis, and subsequently conducted multiple model analyses with statistically significant variables. Collinearity analysis was performed on all the independent variables before the multiple logistic regression analysis, and independent variables with a variance inflation factor less than 10 were included in the model. We controlled for the effect of other confounders to assess the effect of one factor on the prevalence of myopia. Statistical analyses and data visualization were performed by R software (version 4.1.0), and statistical significance was set at *p* < 0.05.

## Results

**FIGURE 1:**
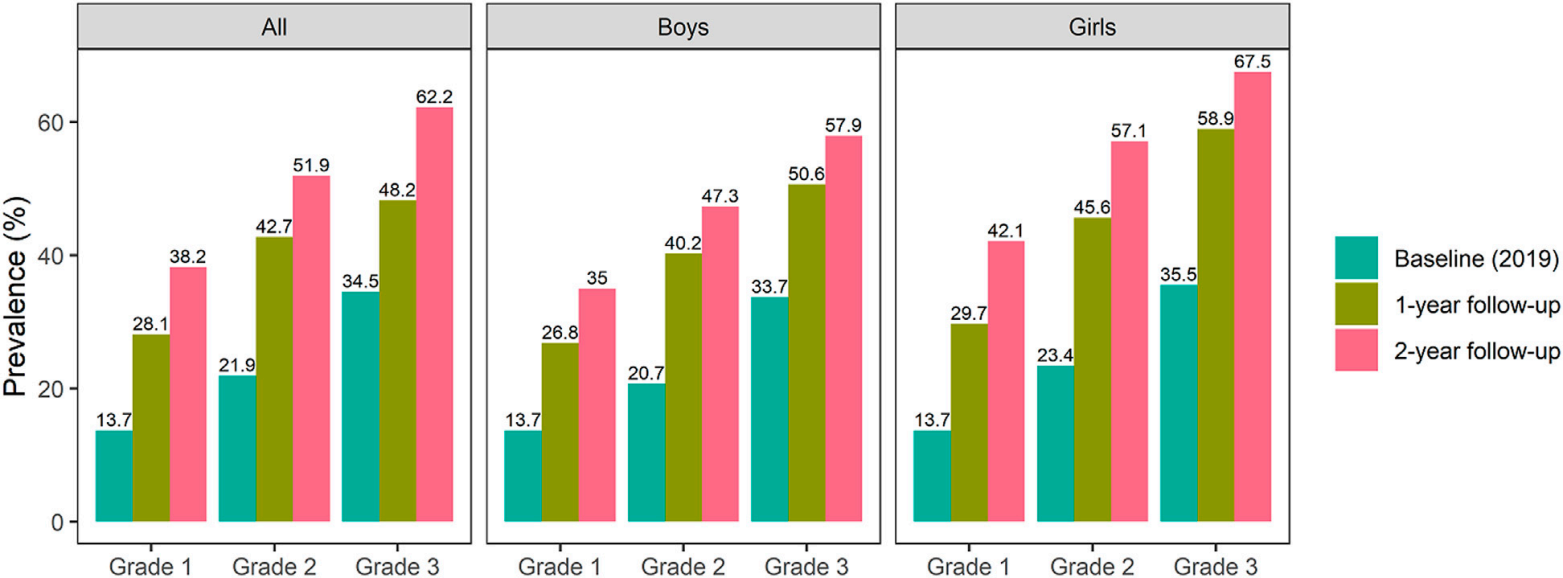
Prevalence of myopia among primary school students in grades 1–3 according to sex (Shenzhen, China. 2019–2021).

The prevalence of myopia in 2019, 2020, and 2021 was 23.4%, 41.9%, and 51.9%, respectively (*χ* ^*2*^ *=* 1257.34, *p <* 0.001)*.* The baseline prevalence of myopia among students in grades 1, 2, and 3 was 13.7%, 21.9%, and 34.5%, respectively, in 2019% and 38.2%, 51.9%, and 62.2%, respectively, after the 2-year follow-up. The baseline prevalence of myopia among boys in grades 1, 2, and 3 was 13.7%, 20.7%, and 33.7% in 2019, respectively, and 35.0%, 47.3%, and 57.9%, respectively, after the 2-year follow-up. The baseline prevalence of myopia among girls in grades 1, 2, and 3 was 13.7%, 23.4%, and 35.5%, respectively, in 2019% and 42.1%, 57.1%, and 67.5%, respectively, after the 2-year follow-up (Table 1; Figure 1). The prevalence of myopia among boys and girls increased gradually over time (*p <* 0.001).

**TABLE 1:** Incidence and prevalence of myopia among students in grades 1–3 (Shenzhen, China. 2019–2021).

	Incidence (%)		*χ* ^*2*^	*P*	Prevalence (%)			*χ* ^*2*^	*P*
	1-year follow-up (2020)	2-year follow-up (2021)			Baseline (2019)	1-year follow-up (2020)	2-year follow-up (2021)		
Grade 1									
All (*n* = 2,409)	22.5	20.6	1.87	0.17	13.7	28.1	38.2	373.30	<0.001
Boys (*n* = 1,299)	20.7	16.2	6.60	0.01	13.7	26.8	35.0	52.67	<0.001
Girls (*n* = 1,110)	24.5	26.0	0.47	0.49	13.7	29.7	42.1	231.67	<0.001
Grade 2									
All (*n* = 2,713)	31.5	23.8	25.40	<0.001	21.9	42.7	51.9	539.60	<0.001
Boys (*n* = 1,464)	28.8	19.7	21.14	<0.001	20.7	40.2	47.3	251.45	<0.001
Girls (*n* = 1,249)	34.9	29.2	5.55	0.02	23.4	45.6	57.1	301.20	<0.001
Grade 3									
All (*n* = 2,475)	35.9	24.4	39.24	<0.001	34.5	48.2	62.2	384.63	<0.001
Boys (*n* = 1,360)	30.8	21.0	18.16	<0.001	33.7	50.6	57.9	170.42	<0.001
Girls (*n* = 1,115)	42.3	29.4	18.62	<0.001	35.5	58.9	67.5	249.37	<0.001
Grades 1–3	29.5	22.7	52.43	<0.001	23.4	41.9	51.9	1257.34	<0.001
Boys (*n* = 4,123)	26.5	19.4	40.73	<0.001	22.8	39.4	46.9	554.30	<0.001
Girls (*n* = 3,474)	33.1	31.5	1.37	0.24	24.2	44.8	55.6	730.75	<0.001

For grade 1 students, the prevalence of mild, moderate, and high myopia was 13.0%, 0.6%, and 0.2% at baseline, respectively, and it increased to 33.4%, 4.6%, and 0.2% after the 2-year follow-up, respectively. For grade 2 students, the prevalence of mild, moderate, and high myopia was 20.1%, 1.8%, and 0.1% at baseline, and it increased to 41.6%, 9.5%, and 0.8% after the 2-year follow-up, respectively. For grade 3 students, the prevalence of mild, moderate, and high myopia was 29.8%, 4.4%, and 0.3% at baseline, and increased to 45.7%, 15.3%, and 1.2% after the 2-year follow-up, respectively. The prevalence of mild, moderate and high myopia among students from grades 1–3 increased with the increase in grade (Supplementary Figure S1).

The incidences of myopia among the students in this study were 29.5% and 22.7% during 1- and 2-year follow-up, respectively; the incidence was higher in the former year than in the latter year (*χ* ^*2*^ *=* 52.43, *p* < 0.001). The incidences of myopia were 22.5%, 31.5%, and 35.9% in 2020 for grades 1, 2, and 3 students, respectively**,** and 20.6%, 23.8%, and 24.4% in 2021, respectively (Table 1). The SERs of the right eye among primary school students in 2019, 2020, and 2021 were −0.40 D, −0.87 D, and −1.12 D, respectively. The SERs of the right eye among grades 1, 2, and 3 were −0.15 D, −0.36 D, and −0.70 D in 2019, respectively, which decreased to −0.76 D, −1.12 D, and −1.49 D, respectively, in 2021. The 2-year progression of the SER was 0.61D, 0.76D, and 0.79D for grade 1, grade 2 and grade 3, respectively. The change in the SER among students in 2020 was significantly higher than that in 2021 (Table 2).

**TABLE 2:** Change of the SER (D) in students from grades 1–3 (Shenzhen, China. 2019–2021).

	SER			F*	*P*	Change of the SER		*t*	*P*
	Baseline (2019)	1-year follow-up (2020)	2-year follow-up (2021)			1-year follow-up (2020)	2-year follow-up (2021)		
Grade 1									
All (*n* = 2,409)	−0.15 (1.02)	−0.51 (1.13)	−0.76 (1.29)	591.41	<0.001	−0.35 (1.07)	−0.25 (0.90)	−3.56	<0.001
Boys (*n* = 1,299)	−0.17 (1.11)	−0.46 (1.19)	−0.71 (1.35)	5.14	<0.001	−0.29 (1.09)	−0.25 (0.86)	−0.91	0.04
Girls (*n* = 1,110)	−0.13 (0.90)	−0.56 (1.06)	−0.82 (1.21)	7.81	<0.001	−0.43 (1.04)	−0.25 (0.95)	−4.24	<0.001
Grade 2									
All (*n* = 2,713)	−0.36 (1.05)	−0.88 (1.34)	−1.12 (1.50)	735.81	<0.001	−0.52 (1.03)	−0.24 (0.87)	−10.76	<0.001
Boys (*n* = 1,464)	−0.32 (1.05)	−0.83 (1.34)	−1.05 (1.52)	105.33	<0.001	−0.51 (0.96)	−0.22 (0.83)	−8.96	<0.001
Girls (*n* = 1,249)	−0.41 (1.05)	−0.93 (1.34)	−1.20 (1.48)	94.91	<0.001	−0.52 (1.10)	−0.26 (0.91)	−6.31	<0.001
Grade 3									
All (*n* = 2,475)	−0.70 (1.31)	−1.20 (1.52)	−1.49 (1.70)	840.74	<0.001	−0.52 (1.02)	−0.27 (0.79)	−9.81	<0.001
Boys (*n* = 1,360)	−0.66 (1.33)	−1.15 (1.54)	−1.41 (1.74)	116.43	<0.001	−0.50 (1.03)	−0.25 (0.72)	−7.66	<0.001
Girls (*n* = 1,115)	−0.74 (1.28)	−1.27 (1.49)	−1.59 (1.65)	102.18	<0.001	−0.55 (1.08)	−0.30 (0.87)	−6.25	<0.001
Grades 1–3									
All (*n* = 7,597)	−0.40 (1.15)	−0.87 (1.38)	−1.12 (1.54)	271.93	<0.001	−0.47 (1.04)	−0.25 (0.85)	−14.28	<0.001
Boys (*n* = 4,123)	−0.38 (1.18)	−0.82 (1.40)	−1.06 (1.57)	130.37	<0.001	−0.44 (1.02)	−0.24 (0.81)	−9.86	<0.001
Girls (*n* = 3,474)	−0.43 (1.12)	−0.93 (1.35)	−1.20 (1.49)	114.80	<0.001	−0.50 (1.08)	−0.27 (0.91)	−9.60	<0.001

Data are presented as mean (standard deviation). SER, spherical equivalent refraction. *, repeated measures analysis of variance.

The 2-year cumulative incidences of myopia among primary school students with a baseline SER of 0.00 D to −0.50 D, +0.50 D to 0.00 D, +1.00 D to +0.50 D, +1.50 D to +1.00 D, and > +1.50 D were 54.1%, 36.3%, 15.5%, 10.1%, and 2.5% after the 2-year follow-up, respectively. The incidence of myopia decreased significantly after the 2-year follow-up with an increase in the baseline SER (*χ* ^*2*^ *=* 420.40, *p* < 0.001) (Table 3).

**TABLE 3:** 2-year cumulative incidence of myopia among primary school students according to the baseline SER (Shenzhen, China. 2019–2021).

Baseline SER (D)	All				Boys				Girls			
	n	Incidence, %	*χ* ^*2*^ *	*P*	n	Incidence, %	*χ* ^*2*^ *	*P*	n	Incidence, %	*χ* ^*2*^ *	*P*
−0.5 < SER ≤0	2,060	54.1	420.40	<0.001	1,112	49.2	229.21	<0.001	948	59.7	190.20	<0.001
0 < SER ≤0.5	1,949	36.3			1,102	31.6			847	42.4		
0.5 < SER ≤1	607	15.5			343	11.7			264	20.5		
1 < SER ≤1.5	109	10.1			60	10.0			49	10.2		
SER >1.5	80	2.5			49	0.0			31	6.5		

SER, spherical equivalent refraction; D, diopters; *, Chi square test for trend.

The demographic characteristics of participants in this study is shown in Table 4. The prevalence of myopia was higher among girls than boys (*χ* ^*2*^ *=* 30.56, *p* < 0.001). The prevalence of myopia among students with myopic parents (*χ* ^*2*^ *=* 72.60, *p* < 0.001), digital device exposure for >1 h per day (*χ* ^*2*^ *=* 64.22, *p* < 0.001), <9 h of sleep per day (*χ* ^*2*^ *=* 264.52, *p* < 0.001), who spent less time performing outdoor activities per day (*t =* 8.14, *p* < 0.001), with a lower baseline SER (*t =* 19.48, *p* < 0.001), older age (*t =* −12.10, *p* < 0.001), and higher parental education (*χ* ^*2*^ *=* 4.71, *p* < 0.001) was higher than that of students without myopic parents, digital device exposure for <1 h per day, >9 h of sleep per day, who spent more time performing outdoor activities per day, had a higher baseline SER, younger age, and lower parental education, respectively.

**TABLE 4:** Characteristics of students in the present study in 2021 (Shenzhen, China. 2019–2021).

Characteristic	No myopia (*n* = 3,654)	Myopia (*n* = 3,943)	Statistical value	*P*
Sex, n (%)				
Boy	2,103 (51.0)	2,020 (49.0)	*χ* ^*2*^ = 30.56	<0.001
Girl	1,551 (44.6)	1,923 (55.4)		
Age, mean (SD), years	9.21 (2.18)	11.41 (1.94)	*t* = −12.10	<0.001
Parental myopia, n (%)				
0 parent	2,420 (51.4)	2,288 (48.6)	*χ* ^*2*^ = 72.60	<0.001
1 parent	843 (45.8)	999 (54.2)		
2 parents	391 (37.3)	656 (62.7)		
Time of birth, n (%)				
Premature infant	254 (45.8)	300 (54.2)	*χ* ^*2*^ = 1.21	0.27
Normal term infant	3,400 (48.3)	3,643 (51.7)		
Parental education, n (%)				
High school and below	1,101 (50.0)	1,099 (50.0)	*χ* ^*2*^ = 4.71	0.03
Junior college and above	2,553 (47.3)	2,844 (52.7)		
Average monthly income, yuan, n (%)				
<10,000	1,862 (49.3)	1,917 (50.7)	*χ* ^*2*^ = 3.69	0.16
10,000–49,999	1,470 (47.1)	1,651 (52.9)		
≥50,000	332 (47.0)	375 (53.0)		
Average living space, square meters, n (%)				
<20	1,295 (48.9)	1,354 (51.1)	*χ* ^*2*^ = 3.08	0.38
20–29	1,004 (46.4)	1,160 (53.6)		
30–39	704 (47.5)	779 (52.5)		
≥40	626 (48.1)	675 (51.9)		
Sleep time per day, hours (%)				
<8	763 (34.8)	1,432 (65.2)	*χ* ^*2*^ = 264.52	<0.001
8–9	2,190 (51.3)	2,078 (48.7)		
>9	711 (62.2)	433 (37.8)		
Digital device exposure per day, hours (%)				
<0.5	1,444 (53.7)	1,243 (46.3)	*χ* ^*2*^ = 64.22	<0.001
0.5–1	1,325 (47.1)	1,490 (52.9)		
>1	885 (42.2)	1,210 (57.8)		
Time spent performing outdoor activities per day, mean (SD), hours	2.68 (1.47)	1.14 (1.01)	*t* = 8.14	<0.001
Baseline SER of the right eye, mean (SD), D	−0.08 (0.81)	−2.45 (1.75)	*t* = 19.48	<0.001

SD, standard deviation; SER, spherical equivalent refraction; D, diopters.

**FIGURE 2:**
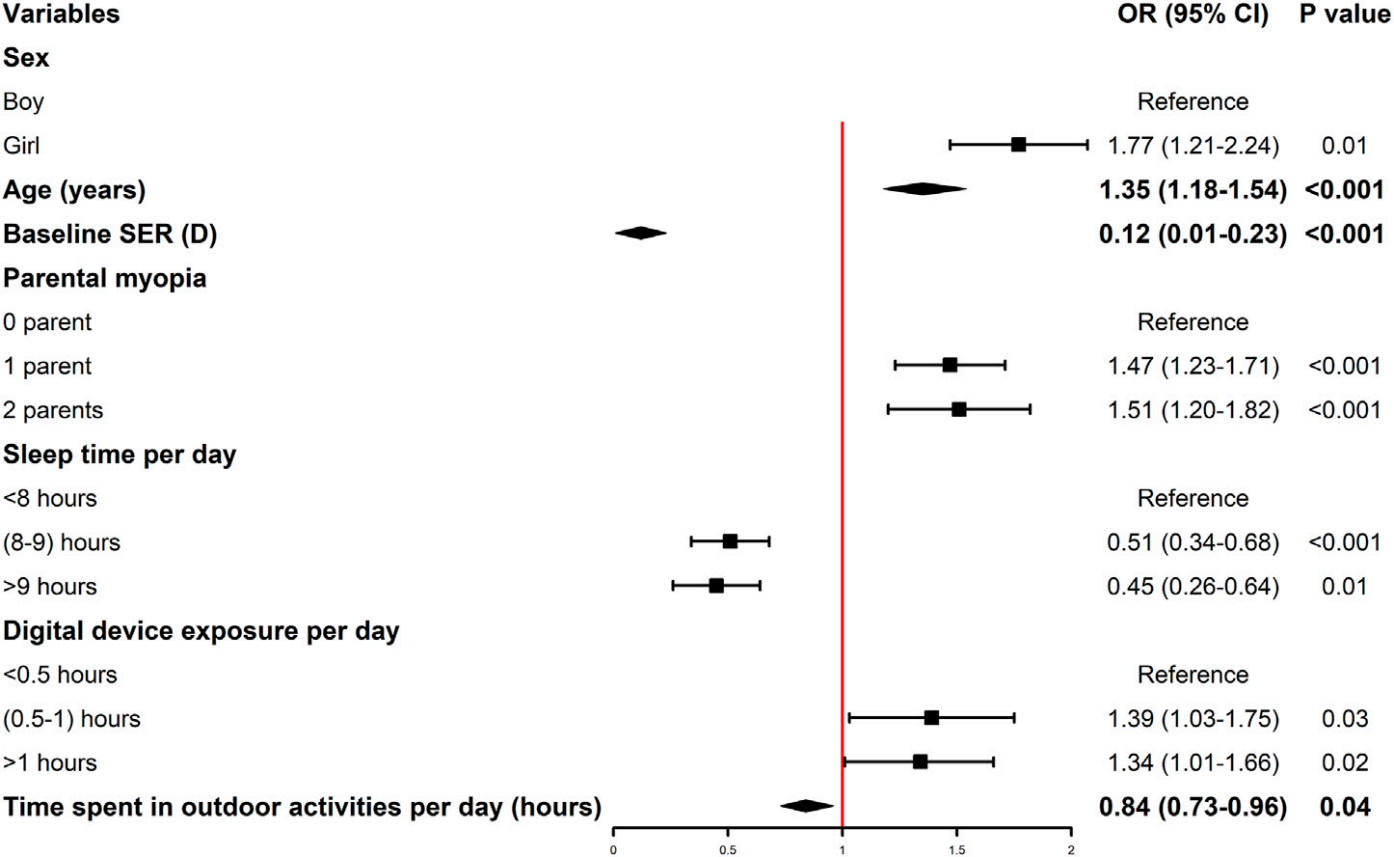
ORs and 95% CIs of myopia according to the influencing factors (Shenzhen, China. 2019–2021). OR, odds ratio; CI, confidence interval; SER, spherical equivalent refraction; D, diopters. *, The OR values of quantitative variables are represented by diamond legend, while those of qualitative variables are represented by square legend. The 95% CI of the OR of the quantitative variable is represented by the leftmost and rightmost ends of the diamond.

A multiple logistic regression model was constructed to analyze the influencing factors of myopia. According to the OR and 95% CI, students with myopia were girl (OR = 1.77, 95% CI: 1.21–2.24), older (OR = 1.35, 95% CI: 1.18–1.54), had a lower baseline SER (OR = 0.12, 95% CI: 0.01–0.23), had one parent with myopia (OR = 1.47, 95% CI: 1.23–1.71), had two parents with myopia (OR = 1.51, 95% CI: 1.20–1.82), slept >9 h per day (OR = 0.45, 95% CI: 0.26–0.64), slept 8–9 h per day (OR = 0.51, 95% CI: 0.34–0.68), spent less time performing outdoor activities (OR = 0.84, 95% CI: 0.73–0.96), spent >1 h per day using a digital device (OR = 1.39, 95% CI: 1.03–1.75), and spent 0.5–1 h per day using a digital device (OR = 1.34, 95% CI: 1.01–1.66) (Figure 2). For each 1 year increase in age, the risk of myopia in older students was 1.35 times that of younger students. For each 1D increase in baseline SER, the risk of myopia in students with higher baseline SER was 0.12 times that of those with lower baseline SER. For each 1 h increase in time spent in outdoor activities per day, the risk of myopia among those who spent more time in outdoor activities was 0.84 times that of those who spent less time in outdoor activities.

## Discussion

We investigated the prevalence and incidence of myopia and change in SER among students in grades 1–3 from 2019 to 2021 and explored the influencing factors of myopia in this study. Myopia developed early in primary school students in Shenzhen, and the incidence of myopia was high, especially during the coronavirus disease 2019 (COVID-19) pandemic. The prevalence of myopia among students increased annually, and the incidence of myopia was higher in 2020 than in 2021. Sex, age, parental myopia, digital device exposure per day, baseline SER, sleep time per day, and time spent performing outdoor activities per day were the potential factors influencing myopia.

Many studies have demonstrated myopia to be related to sex of the individual (29, 30). In the present study, the prevalence of myopia among girls was higher than that among boys. Therefore, girls were not only more prone to myopia compared to boys but also a worse degree of myopia. The Anyang cohort study found that the incidence of myopia among boys in primary school was lower than that among girls (24). Moreover, the prevalence of myopia among boys in Poland was lower than that among girls (31). Eighteen-year-old Caucasian and East Asian girls were twice as likely to be myopic compared with 18-year-old boys; however, there was no significant difference between different sexes among Latinos, South Asians, and Hispanics (32). Myopia is also related to factors other than sex. This could be owing to the following reasons: First, girls tend to undergo puberty earlier compared to boys, and their axis grows faster, which can result in an increase in the prevalence of myopia. Second, girls prefer low-level physical activities, such as reading and writing, resulting in more near work and a lack of outdoor activities, which leads to an increase in the prevalence of myopia (33). At present, the relationship between myopia and sex among different populations is inconsistent. Further studies are warranted owing to the complex etiology of myopia.

The incidence of myopia and change in SER among primary school students in Shenzhen increased with the increase of grade level, which is consistent with the findings of another study (34). In the present study, both the incidence of myopia and the change in SER in 2020 were much higher than that in 2021. However, in the present study, the higher incidence in 2020 than in 2021 was not similar among girls in grade 1, possibly because they had higher hyperopia reserves than boys (−0.13 D vs. −0.17 D). Although the incidence of myopia and change in SER can be obtained directly from longitudinal examination in the same population, the effects of physiological refractive development on the change in the incidence and SER between the two time points should be considered. The annual incidence of myopia in primary school students has been shown to increase with age (24). Moreover, the annual change in SER in primary school students first increased and then decreased with the increase of age, and the annual change in SER reached the maximum in grade 3 and 4 students (−0.59 D) (24). The COVID-19 broke out in China in December 2019. To stop the spread of the COVID-19, all the students in China received online education at home and did not receive education in person at school until May 2020 (21). The annual change in the SER among primary school students from grades 1 to 3 during the COVID-19 pandemic (in 2019–2020) in this study was smaller than that in primary school students from grades 1 to 3 in Shanghai in 2010–2015 (33). The prevalence of myopia among students increased and the progression of myopia accelerated during the COVID-19 pandemic (21, 34, 35). To prevent and control the COVID-19 pandemic in 2020, students attended classes at home, which led to a decrease in the children’s physical activities and an increase in the use of electronic devices (36). Therefore, the lifestyle changes attributed to the COVID-19 pandemic may have resulted in a higher incidence and larger SER changes in the 1-year follow-up compared to the 2-year follow-up. The annual incidence of myopia among students in primary school in this study was higher than that in Australia (<2.2%) (25), Singapore (14.2%) (37), Anyang (20.0%–30.0%) (38), and Chongqing (10.6%) (22), but was close to that in Guangzhou (20.0%–30.0%) (23). Myopia screening by non-cycloplegic refraction was conducted in this study, which was consistent with that in Guangzhou (23) and but not with that in Australia (25), Singapore (37), Anyang (38), and Chongqing (22). However, myopia screening by non-cycloplegic refraction cannot identify pseudomyopia, which may result in the overestimation of the prevalence of myopia (38).

Eyes are in a hyperopic state (+2.50 to +3.00 D) at birth. With the growth and development of the eye, the eye tends to become slightly hyperopic or emmetropic, i.e., emmetropization (39). The refractive D of hyperopia is called the hyperopia reserve (38). In the present study, with the increase in the hyperopia reserve, the cumulative incidence of myopia in primary school students decreased during the 2-year follow-up period. The 2-year cumulative incidence of myopia among students with an SER of −0.50 D to 0.00 D can be as high as 57.6%, and <20% with an SER > +1.00 D. Previous studies have suggested that an increase in the early intervention efforts for primary school students whose hyperopia reserve is < +1.00 D could prevent rapid consumption of the hyperopia reserve.

The risk of myopia is associated with parental myopia according to previous studies (40, 41). Moreover, we found parental myopia have a great influence on myopia in children. Studies have identified more than 150 single-nucleotide polymorphisms associated with myopia (42). Consistent with previous studies (40), students who spent more time performing outdoor activities were less likely to become myopic. Previous studies have demonstrated that exposure to high-intensity sunlight can slow axial growth (43, 44), and may stimulate dopamine synthesis and release (45). Digital devices, such as computers and smartphone, have become exceeding popular in recent years, and one-third of the children aged 1**–**6 use mobile phones for 1**–**2 h per day (46). The prevalence of myopia increases with the popularity of digital devices (47), and exposure to digital devices can increase the risk of myopia (48). Consistent with previous studies (47, 48), students who spent more time using electronic devices were more likely to become myopic. Previous studies have confirmed that the SER increases by 0.10 D with a 1 h/day increase in sleep, and students who slept for more than 9 h/day had a 40% lower risk of developing myopia than those who slept for less than 5 h/day (16). Overall, the lack of sleep could be an important risk factor of myopia (49). In accordance with previous studies, we found that students who spent more time sleeping were less likely to become myopic. Furthermore, previous studies have shown that a higher risk of myopia was associated with higher family income (50, 51), and children living in areas with a higher population density have a higher risk of myopia (52–54). Moreover, the incidence of myopia in premature infants was as high as 75.5% (55). Contrary to previous studies, the prevalence of myopia was not associated with family income, living space, and time of birth in this study.t

The strengths of our study are as follows. The participants in this study were primary school students in grades 1**–**3, which reflect the epidemiological characteristics among children and adolescents in the early education stage. Thus, we were able to explore the development of refraction and myopia early, and provide a scientific basis for the prevention and control of myopia. However, this study has some limitations. First, myopia screening was performed by non-cycloplegic refraction, which cannot identify pseudomyopia and may overestimate the prevalence of myopia. The accuracy of cycloplegic refraction is high and widely used in clinical diagnosis. We should use cycloplegia to paralyze the ciliary muscle of the eye to obtain an accurate refractive status. Since cycloplegia could cause side effects (56), paralyzing the ciliary muscle in all the participants for myopia screening in large populations is challenging. Therefore, many studies use non-cycloplegic refraction to conduct myopia screening. Studies have confirmed the sensitivity and specificity of myopia screening in children aged 6–12 years (85.06% and 89.74%, respectively) who underwent a combination of distant uncorrected visual acuity and non-cycloplegic auto-refraction (57). Second, all the risk factor data in the present study were obtained from a questionnaire, which was subjective. Considering the retrospective design of the investigation, the results are particularly susceptible to recall bias. Past behavior or exposure history relies on the participant’s recollection, but certain details could be challenging to recall accurately. Third, some studies found that biometric indicators, such as the axial length (58) and corneal curvature (59), are associated with refraction status, and biometric indicators were not collected in this study. Fourth, the follow-up duration was short (2 years); thus**,** we may have not comprehensively analyzed the epidemiological characteristics of myopia.

### Conclusion

In conclusion, we found that the prevalence of myopia among primary school students increased annually. The incidence of myopia and the progression of SER in 2020 were higher than that in 2021. The 2-year progression of the SER was 0.61D, 0.76D, and 0.79D for grades 1, 2, and 3, respectively. The prevalence of myopia among girls were higher than that among boys. In accordance with the Implementation Plan for Comprehensive Prevention and Control of Myopia in Children and Adolescents in China (60), the prevalence of myopia among primary school students is anticipated to be regulated to within 38% by 2030. Thus, interventions for myopia in primary school students should be strengthened, especially in the lower grades. Establishing good habits concerning eye use, encouraging outdoor activities, maintaining adequate sleep, and monitoring hyperopia reserve among primary school students are crucial for the prevention of myopia.
