# Supplementary figures and images for: Epidemiological Characteristics and Influencing Factors of Myopia Among Primary School Students in Southern China: A Longitudinal Study
Source: Int J Public Health. 2023 Feb 14;68:1605424. doi: 10.3389/ijph.2023.1605424 (PMC9971006; doi:10.3389/ijph.2023.1605424)

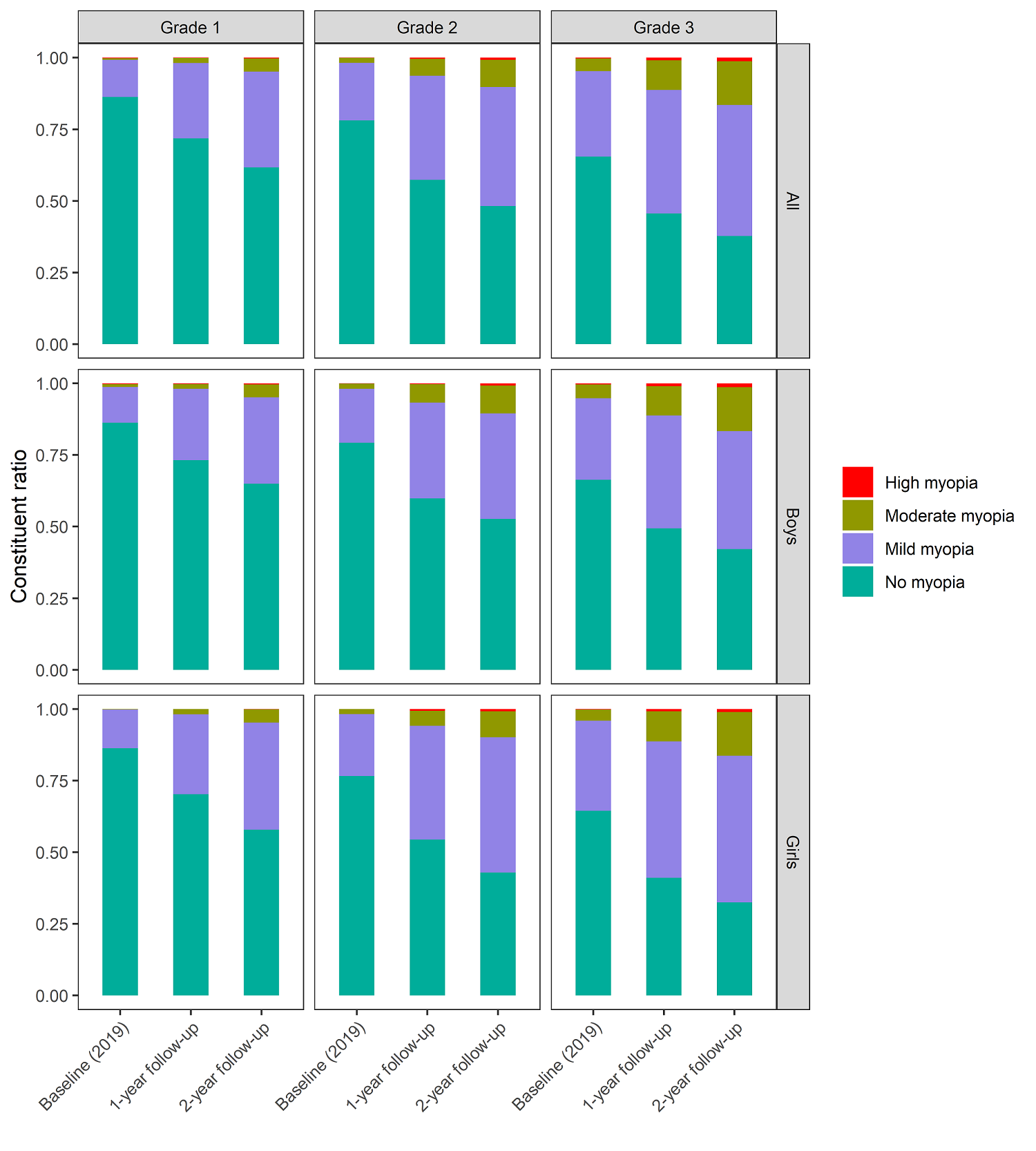

Supplement: Supplementary file 1 [file Image1.TIFF]
